# IL-22-Producing RORγt-Dependent Innate Lymphoid Cells Play a Novel Protective Role in Murine Acute Hepatitis

**DOI:** 10.1371/journal.pone.0062853

**Published:** 2013-04-23

**Authors:** Atsuhiro Matsumoto, Takanori Kanai, Yohei Mikami, Po–Sung Chu, Nobuhiro Nakamoto, Hirotoshi Ebinuma, Hidetsugu Saito, Toshiro Sato, Hideo Yagita, Toshifumi Hibi

**Affiliations:** 1 Division of Gastroenterology and Hepatology, Department of Internal Medicine, Keio University School of Medicine, Tokyo, Japan; 2 Department of Microbiology and Immunology, Keio University School of Medicine, Tokyo, Japan; 3 Department of Immunology, Juntendo University School of Medicine, Tokyo, Japan; McGill University, Canada

## Abstract

Retinoid-related orphan receptor (ROR) γt is known to be related to the development and function of various immunological compartments in the liver, such as Th17 cells, natural killer T (NKT) cells, and innate lymphoid cells (ILCs). We evaluated the roles of RORγt-expressing cells in mouse acute hepatitis model using RORγt deficient (RORγt^−/−^) mice and RAG-2 and RORγt double deficient (RAG-2^−/−^ × RORγt^−/−^) mice. Acute hepatitis was induced in mice by injection with carbon tetrachloride (CCl_4_), to investigate the regulation of liver inflammation by RORγt-expressing cells. We detected *RORC* expression in three compartments, CD4^+^ T cells, NKT cells, and lineage marker-negative SCA-1^+^Thy1^high^ ILCs, of the liver of wild type (WT) mice. CCl_4_-treated RORγt^−/−^ mice developed liver damage in spite of lack of RORγt-dependent cells, but with reduced infiltration of macrophages compared with WT mice. In this regard, ILCs were significantly decreased in RAG-2^−/−^ × RORγt^−/−^ mice that lacked T and NKT cells. Surprisingly, RAG-2^−/−^ × RORγt^−/−^ mice developed significantly severer CCl_4_-induced hepatitis compared with RAG-2^−/−^ mice, in accordance with the fact that hepatic ILCs failed to produce IL-22. Lastly, anti-Thy1 monoclonal antibody (mAb), but not anti-NK1.1 mAb or anti-asialo GM1 Ab administration exacerbated liver damage in RAG-2^−/−^ mice with the depletion of liver ILCs. Collectively, hepatic RORγt-dependent ILCs play a part of protective roles in hepatic immune response in mice.

## Introduction

Retinoid-related orphan receptor γt (RORγt) is a transcription factor that regulates a variety of immunological processes [1]–[3] and has an indispensable role in the development of Th17 cells [1]. Activated Th17 cells secrete a variety of IL-17 family cytokines including IL-17A, IL-21, and IL-22, which promotes tissue inflammation by induction of other proinflammatory mediators and the recruitment of leukocytes to sites of inflammation [4]. Among IL-17 family cytokines, the role of IL-22 in inflammatory responses is unclear owing to contrary data suggesting pro- or anti-inflammatory functions in distinct tissues [5]–[9]. However, during the pathogenesis of acute hepatitis models, IL-22 produced by Th17 cells is thought to have a protective role by preventing tissue injury [7]–[9].

The development of all T cells and NKT cells depend on RORγt to some extent, as it is expressed by CD4 and CD8 double positive thymocytes [10], [11]. Con A-induced acute hepatitis is a lymphocyte-mediated hepatitis model in rodents [12], largely dependent on NKT cell secretion of IFN-γ, TNF-α, and IL-4 [13]–[16].

Furthermore, RORγt is essential for generation of lymphoid tissue inducer (LTi) cells, which are critically involved in the development of secondary lymphoid tissues, such as lymph nodes, Peyer's patches, and cryptopatches [3]. Recent studies reported that various subtypes of RORγt^+^ innate lymphoid cells (ILCs), including LTi cells, producing IL-17A, IL-22 and/or IFN-γ have various roles in innate immune responses, lymphoid tissue formation, and tissue remodeling [2], [17].

A1though the roles of RORγt-dependent Th17 cells or NKT cells in the development of murine acute hepatitis models have been clarified, those of RORγt dependent ILCs have not been investigated. To clarify the roles of RORγt-dependent ILCs in the development of acute hepatitis, we induced CCl_4_-hepatitis in RORγt^−/−^ and RORγt^−/−^ ×RAG-2^−/−^ mice.

## Materials and Methods

### Mice

Eight- to 12-wk-old C57BL/6 (WT) mice were purchased from Japan CLEA (Tokyo, Japan). C57BL/6 background RAG-2-deficient mice were obtained from Central Laboratories for Experimental Animals (Kawasaki, Japan). Mice with green fluorescent protein reporter complementary DNA knocked-in at the site for initiation of RORγt translation on the C57BL/6 background (RORγt^−/−^) were kindly provided by Dr. D. Littman [18]. RAG-2^−/−^ × RORγt^−/−^ mice were obtained by crossing RAG-2^−/−^ mice with RORγt^−/−^ mice. Mice were maintained under specific pathogen-free conditions in the Animal Care Facility of Keio University School of Medicine. This study was carried out in strict accordance with the recommendations in the Guide for the Care and Use of Laboratory Animals of the National Institutes of Health. The protocol was approved by the Committee on the Ethics of Animal Experiments of Keio University School of Medicine. All surgery was performed under anesthesia, and all efforts were made to minimize suffering.

### Preparation of hepatic mononuclear cells

Hepatic mononuclear cells (MNCs) were isolated from the liver as described previously [19]. Briefly, livers were perfused through the portal vein with PBS, then minced and passed through nylon mesh. The filtrate was centrifuged at 50×*g* for 50 seconds and supernatant was collected and centrifuged. Cells were suspended in a Hanks' balance salt solution and overlaid on a Histopaque solution (Sigma–Aldrich, St. Louis, MO, USA). After centrifugation at 2000 rpm for 20 minutes, the cells were collected from the upper layer of the Histopaque.

### Induction of CCl_4_ and α-galactosylceramide (αGalCer) hepatitis models

CCl_4_ was purchased from Wako (Osaka, Japan). CCl_4_ (0.75 ml/kg) in olive oil was injected intraperitoneally in each mouse. In some CCl_4_-induced hepatitis experiments, mice were injected intraperitoneally with anti-asialo GM1 Ab (200 μg/mouse; Wako), anti-Thy1 monoclonal antibody (mAb; 250 μg/mouse, clone 30H12) or anti-NK1.1 mAb (250 μg/mouse, clone PK136) 1 day and 3 hours before administration of CCl_4_. Recombinant murine IL-22 (2.5 μg/mouse, Peprotech) was administrated intravenously immediately before administration of CCl_4_.αGalCer (KRN7000) was purchased from Funakoshi Co., Ltd. (Tokyo, Japan). αGalCer (100 μg/kg) was injected intravenously into the tail vein of animals 12 hours before examination.

### Measurement of liver injury

Serum ALT levels were measured using the LDH-UV kinetic method (SRL Inc., Tokyo, Japan). Livers were fixed in 10% formalin and embedded in paraffin. Sections were stained with H&E and examined. Statistic evaluation was performed by using pathological score referring to the previous report [20]. Briefly, the overall degree or grade of necrosis was scored from 0 to 4 on the basis of the severity and distribution of the necrotic lesions and the number of lobes affected as follows: 0– None, 1– Minimal to Mild, 2– Moderate, 3– Marked, 4– Severe to Diffuse.

### Flow cytometric analysis and cell sorting

Flow cytometric analysis was performed as described previously [19]. Briefly, after blocking with anti-FcR (CD16/32, BD Pharmingen, San Jose, CA, USA) for 15 minutes, the cells were incubated with fluorescence-labeled mAb at 4°C for 20 minutes. For intracellular staining, cells were stimulated for 3 h with mouse recombinant IL-23 (40 ng/mL, R&D Systems, Minneapolis, MN, USA). For the final 1.5 h, GolgiPlug (BD Pharmingen) was added. After surface staining, the cells were resuspended in Fixation/Permeabilization solution (BD Pharmingen), and intracellular staining was performed. In terms of intracellular staining for transcriptional factor, Fixation/Permeabilization Concentrate (eBioscience) was used, and intracellular staining was performed without stimulation. The following mAbs were used: anti-CD11b (FITC and PE), anti-CD11c (PE-Cy7), anti-B220 (FITC), anti-Gr-1 (FITC), anti-IL-22 (PE), anti-IL-17A (PE), anti-IFN-γ (PE), anti-NKp46 (PE), anti-IL-7R (Biotin), anti-TCRβ (APC), anti-NK1.1 (PE), anti-SCA-1 (PE-Cy7), anti-Thy1 (APC-Cy7), anti-c-kit (PE), anti-CD25 (PE), anti-CD44 (APC), anti-CCR6 (APC), anti-RORγt (PE), Streptavidin (PE) and 7-AAD from eBioscience (San Diego, CA, USA), BD Pharmingen, and BioLegend (San Diego, CA, USA). The stained cells were analyzed on FACS Canto II (BD) and the data were analyzed using Flowjo software (Tree Star Inc.). Cell sorting was performed on FACSAria™ to obtain a pure population.

### RT-qPCR

RNA was extracted from liver tissues using TRIzol reagent (Invitrogen, Carlsbad, CA, USA). Complementary DNA was synthesized from extracted RNA using TaqMan® Reverse Transcription Reagents (Applied Biosystems, Foster City, CA, USA). Reverse transcription was performed at 25°C for 10 min, 48°C for 30 min, and then 95°C for 5 min. Complementary DNA was analyzed by RT-qPCR using TaqMan® Universal PCR Master Mix (Applied Biosystems) in Applied Biosystems StepOne™/StepOnePlus™ (Applied Biosystems). The following probes were purchased from Applied Biosystems: *Il22* (Mm00444241_m1), *Ifng* (Mm99999071_m1), *Tnf* (Mm99999068_m1), *Il6* (Mm00446190_m1), *Rorc* (Mm01261022_m1) and mouse β-actin. Relative quantification was achieved by normalizing to the value of the β-actin gene.

### Statistical analysis

The results were expressed as the mean ± standard error of mean (SEM). Groups of data were compared using the Student's *t*-test. In terms of the evaluating pathological score, data compared using the Mann-Whitney test. Differences were considered to be statistically significant when *p*<0.05. ***p<0.001, **p<0.01, *p<0.05. NS, not significantly different.

## Results

### CD4^+^ T cells, NKT Cells and ILCs in the Liver Expressed RORγt, and ILCs Were Decreased in RAG-2^−/−^ × RORγt^−/−^ mice

**Figure 1:**
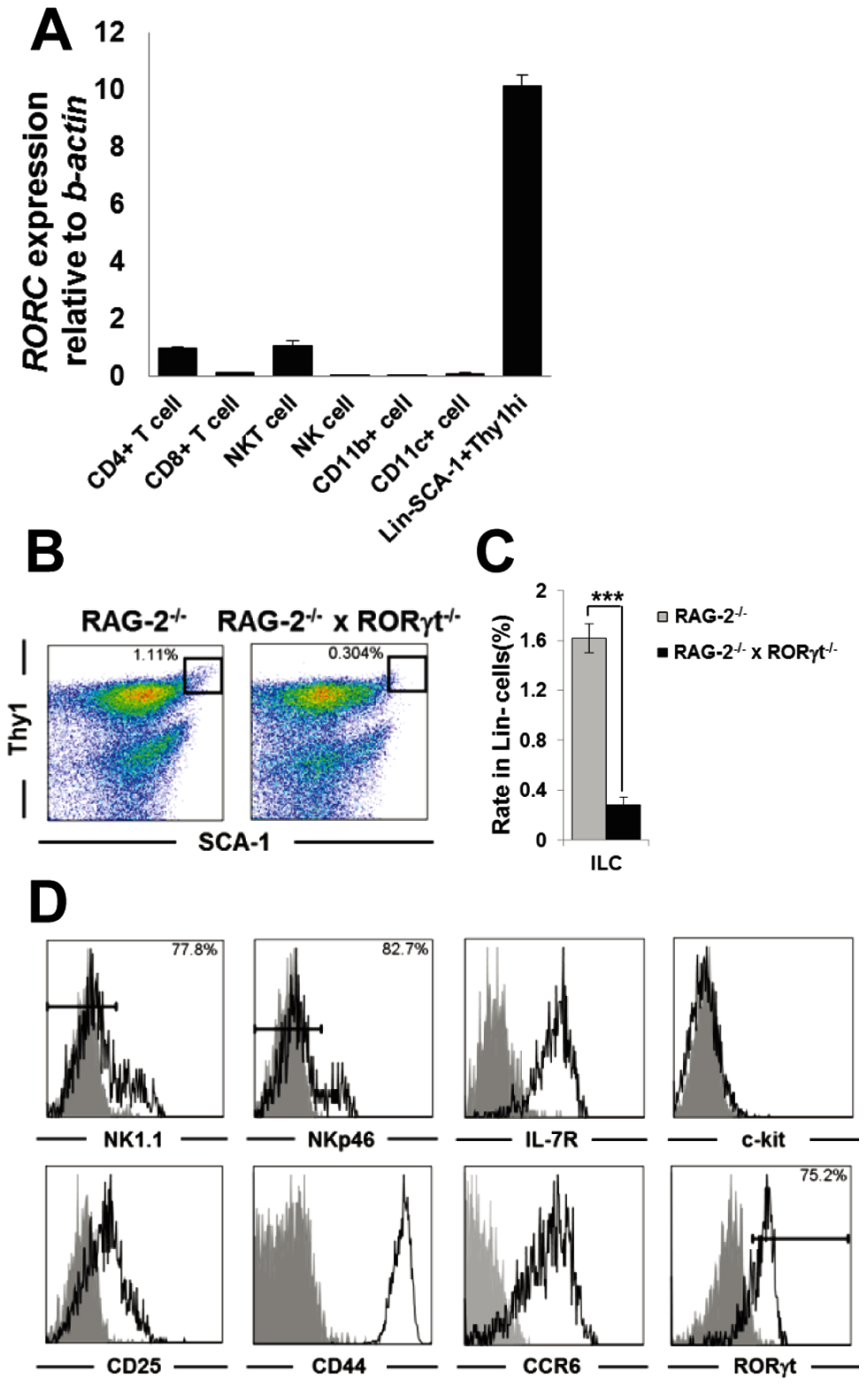
Hepatic ILCs express RORγt and are decreased in RAG-2^−/−^ × RORγt^−/−^ mice. (A) RORγt expression in hepatic MNCs. RORγt mRNA expression in the indicated hepatic MNCs subsets was measured by RT-qPCR and normalized relative to β-actin expression. Error bars represent SEM of triplicate samples. (B) SCA-1/Thy1 staining of the Lin^−^ fraction of hepatic MNCs from RAG-2^−/−^ or RAG-2^−/−^ × RORγt^−/−^ mice. (C) Ratio of SCA-1^+^Thy1^high^ ILCs in Lin^−^ hepatic MNCs from RAG-2^−/−^ or RAG-2^−/−^ × RORγt^−/−^ mice. Data show the mean ± SEM (n = 4/group). (D) Expression of NK1.1, NKp46, IL-7R, c-kit, CD25, CD44, CCR6 and RORγt on hepatic ILCs from RAG-2^−/−^ mice. Data are representative of three independent experiments.

To determine the expression of RORγt in the liver of normal mice, we isolated the following MNC subsets: CD11b^+^TCRβ^−^ macrophages, CD11c^+^TCRβ^−^ dendritic cells (DCs), CD4^+^TCRβ^+^ CD4 T cells, CD8^+^TCRβ^+^ CD8 T cells, NK1.1^+^TCRβ^−^ NK cells, NK1.1^+^TCRβ^+^ NKT cells and Lin^−^SCA-1^+^Thy1^high^ ILCs, and evaluated the level of *RORC* expression by RT-qPCR. To obtain ILCs from the liver, we used anti-CD3ε mAb in addition to the lineage markers [2], to further exclude T and NKT cells. Thus, using the isolated liver cell populations we detected the expression of RORγt in CD4 T cells, NKT cells, and ILCs, and observed the expression of RORγt in ILCs was much higher than in CD4 T cells and NKT cells (Fig. 1A).

We also investigated the frequency of hepatic Lin^−^ ILCs. Since T cells are present in the Lin−SCA-1^+^Thy1^high^ population (data not shown), we compared lymphocyte-lacking RAG-2^−/−^ mice with RAG-2^−/−^ × RORγt^−/−^ mice to exclude the possibility of T cell contamination. Consequently, the frequency of hepatic ILCs in RAG-2^−/−^ × RORγt^−/−^ mice was also significantly reduced compared with RAG-2^−/−^ mice (Fig. 1B and 1C). The ILCs expressed IL-7R, CD25, CD44 and CCR6, but not NK1.1, NKp46 and c-kit (Fig. 1D), suggesting that ILCs in the liver had similar characteristics to ILCs in the intestine [2]. Furthermore, almost all of SCA-1^+^Thy1^high^ hepatic ILCs expressed RORγt (Fig. 1D).

### RORγt^−/−^ Mice Develop Carbon Tetrachloride (CCl_4_)-induced Acute Hepatitis

**Figure 2:**
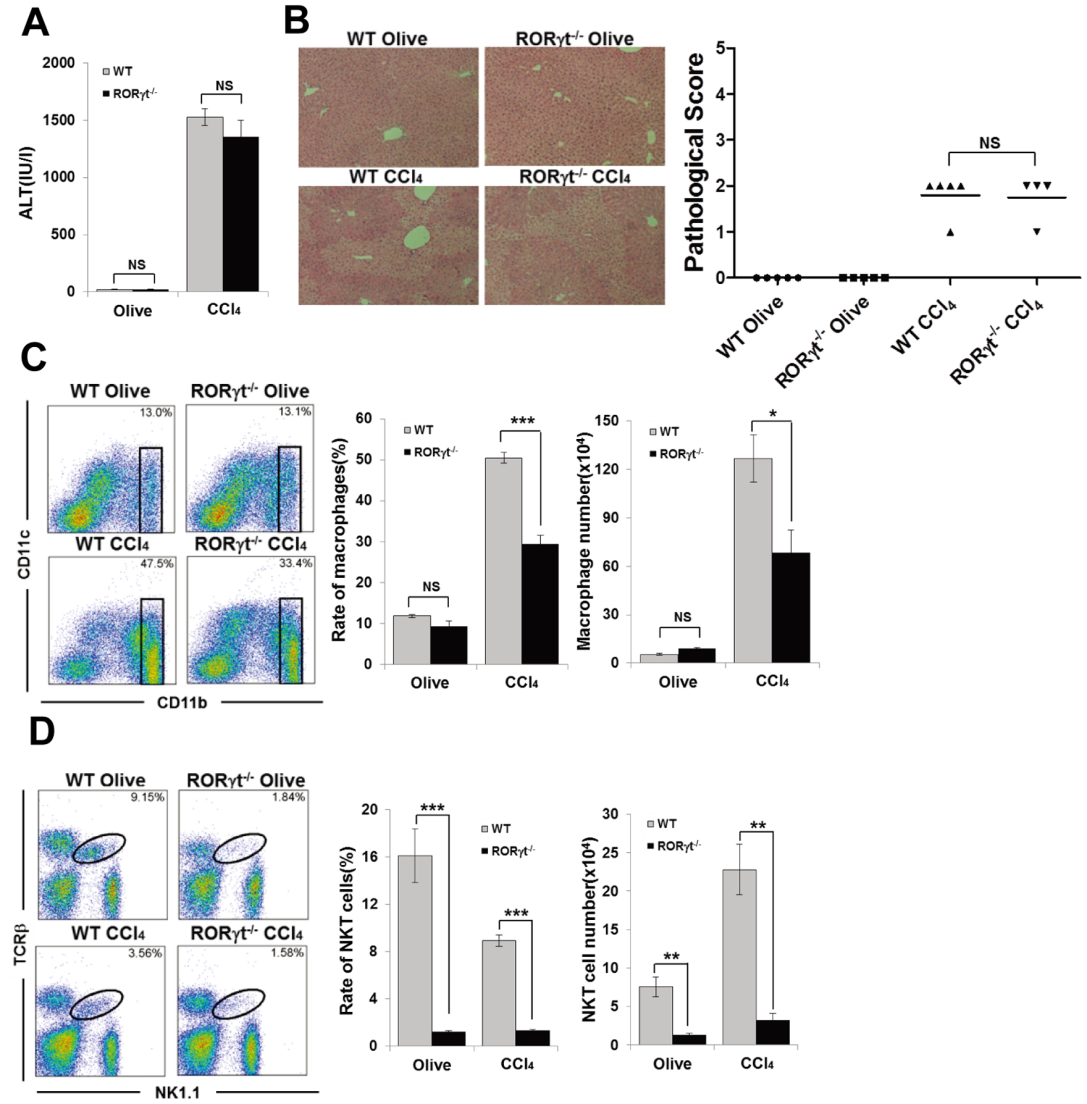
RORγt^−/−^ mice develop CCl_4_-induced hepatitis. (A) Serum ALT levels of WT or RORγt^−/−^ mice 12 h after CCl_4_ (WT n = 5, RORγt^−/−^ n = 4) or olive oil injection (n = 5/group). Data show the mean ± SEM. (B) Representative photomicrographs of H&E-stained liver from each group and pathological score. (C) CD11b/CD11c staining of hepatic MNCs from WT or RORγt^−/−^ mice 12 h after CCl_4_ or olive oil treatment. Ratio and absolute number of CD11b^+^ macrophages in the hepatic MNCs. Data show the mean ± SEM. (D) TCRβ/NK1.1 staining of hepatic MNCs. Ratio and absolute number of NKT cells in the hepatic MNCs. Data show the mean ± SEM. Data are representative of two independent experiments.

To assess the role of RORγt dependent cells including NKT cells, Th17cells and hepatic RORγt^+^ ILCs in hepatitis, we used a carbon tetrachloride (CCl_4_)-induced hepatitis model in WT and RORγt^−/−^ mice. CCl_4_ administration directly damages hepatocytes and induces hepatitis. [21] IL-22 produced by Th17 cells has been reported to act protectively as an epithelial repair factor in the CCl_4_-induced hepatitis model [7]–[9]. However, there was no significant difference in the serum ALT levels between WT and RORγt^−/−^ mice after CCl_4_ treatment (Fig. 2A). Histological assessment showed a similar level of liver injury in CCl_4_-administered WT and RORγt^−/−^ mice (Fig. 2B). Interestingly, although the infiltration of macrophages is essential for the development of this model [19], the rate and number of macrophages were significantly decreased in RORγt^−/−^ mice compared with WT mice after CCl_4_ injection (Fig. 2C). Furthermore, both the frequency and absolute number of NKT cells in the liver of CCl_4_-administered RORγt^−/−^ mice were also significantly lower than those of CCl_4_-administered WT mice (Fig. 2D). We also found that αGalCer, the specific simulator for NKT cells could induce the infiltration of inflammatory macrophage into the liver, and that phenomenon was disappeared in RORγt^−/−^ mice (Fig. S1). The development of the similar degree of hepatitis in RORγt^−/−^ mice following CCl_4_ administration compared with WT mice despite a significant decrease in the number of pathological macrophages and NKT cells indicate the existence of some RORγt-dependent protective cells besides RORγt-dependent pathological NKT cells in this model.

### RORγt Deficiency in RAG-2^−/−^ Mice Exacerbates CCl_4_-induced Hepatitis

**Figure 3:**
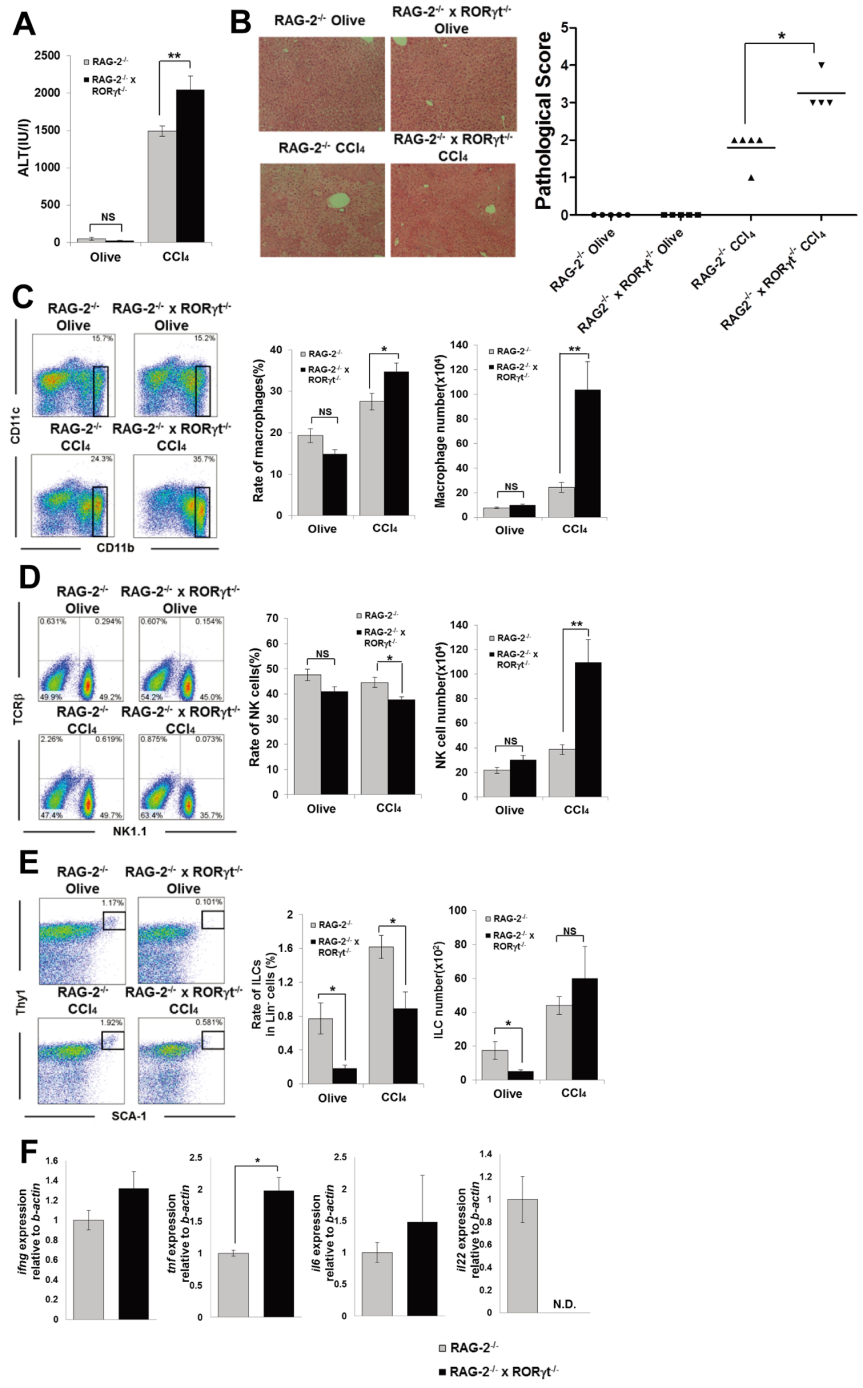
RAG-2^−/−^ × RORγt^−/−^ mice develop more severe CCl_4_-induced hepatitis than RAG-2^−/−^ mice. (A) Serum ALT levels of RAG-2^−/−^ or RAG-2^−/−^ × RORγt^−/−^ mice 12 h after CCl_4_ (RAG-2^−/−^ n = 9, RAG-2^−/−^ × RORγt^−/−^ n = 8) or olive oil injection (n = 8/group). Data show the mean ± SEM. (B) Representative photomicrographs of H&E-stained liver from each group and pathological score. (C) CD11b/CD11c staining of hepatic MNCs from RAG-2^−/−^ or RAG-2^−/−^ × RORγt^−/−^ mice 12 h after CCl_4_ (RAG-2^−/−^ n = 5, RAG-2^−/−^ × RORγt^−/−^ n = 4) or olive oil treatment (n = 5/group). Ratio and absolute number of CD11b^+^ macrophages in the hepatic MNCs. Data show the mean ± SEM. (D) TCRβ/NK1.1 staining of hepatic MNCs. Ratio and absolute number of NK cells in the hepatic MNCs. Data show the mean ± SEM. (E) SCA-1/Thy1 staining of Lin^−^ hepatic MNCs. Ratio of ILCs in Lin^−^ fraction and absolute number of ILCs. (F) Cytokine mRNA expression in the hepatic MNCs from CCl_4_-injected RAG-2^−/−^ or RAG-2^−/−^ × RORγt^−/−^ mice 12 h after CCl_4_ injection was determined by RT-qPCR. Data show the mean ± SEM Cytokine mRNA expression in the hepatic MNCs from CCl_4_-injected RAG-2^−/−^ or RAG-2^−/−^ × RORγt^−/−^ mice 12 h after CCl_4_ injection was determined by RT-qPCR. Data show the mean ± SEM (n = 4/group). Data are representative of three independent experiments.

To investigate the function of RORγt-dependent hepatic ILCs without the impact of NKT and Th17 cells, we next administrated CCl_4_ to RAG-2^−/−^ × RORγt^−/−^ mice. Unlike NKT cell-dependent Con A- or α-galactosylceramide-induced hepatitis model, CCl_4_ administration fully induced hepatitis in RAG-2^−/−^ mice [21]. Surprisingly, unlike that for WT and RORγt^−/−^ mice (Fig. 2A), the serum ALT level was significantly higher in RAG-2^−/−^ × RORγt^−/−^ mice compared with RAG-2^−/−^ mice 12 h after CCl_4_ injection (Fig. 3A). Histological assessment revealed a more severe liver injury in CCl_4_-administered RAG-2^−/−^ × RORγt^−/−^ mice compared with CCl_4_-administered RAG-2^−/−^ mice (Fig. 3B). The frequency and absolute cell number of macrophages was significantly increased in the liver of CCl_4_-administered RAG-2^−/−^ × RORγt^−/−^ mice compared with CCl_4_-administered RAG-2^−/−^ mice (Fig. 3C). Moreover, the absolute cell number of NK cells was also significantly increased in the liver of CCl_4_-administered RAG-2^−/−^ × RORγt^−/−^ mice compared with CCl_4_-administered RAG-2^−/−^ mice owing to the overall increased cell number in the liver (Fig. 3D).

Therefore, we focused on hepatic ILCs in the exacerbation of CCl_4_-induced liver injury as these cells express high levels of RORγt and are significantly decreased in RAG-2^−/−^ × RORγt^−/−^ mice (Fig. 1A). In this regard, it is notable that Buonocore *et al*. reported that Lin^−^SCA-1^+^Thy1^high^ ILCs producing IL-22 resided in *Helicobacter hepaticus*-infected liver [2]. Furthermore, Zenewicz *et al*. previously reported that Con A-induced hepatitis was exacerbated in IL-22 deficient mice, but they emphasized the importance of T cells including Th17 cells as the source of IL-22 in that model [9]. The frequency of Lin^−^SCA-1^+^Thy1^high^ ILCs was significantly lower in RAG-2^−/−^ × RORγt^−/−^ mice after olive oil or CCl_4_ injection, but there was no difference in the number of ILCs after CCl_4_ injection, as the absolute cell numbers of MNCs were markedly increased in CCl_4_-injected RAG-2^−/−^ × RORγt^−/−^ mice owing to the increased severity of disease (Fig. 3E). Interestingly, IL-22 mRNA was detectable only in the hepatic MNCs of CCl_4_-injected RAG-2^−/−^, but not RAG-2^−/−^ × RORγt^−/−^ mice, although they lack Th17 cells (Fig. 3F). Furthermore, the expression of TNF-α mRNA in hepatic MNCs was significantly increased in CCl_4_-injected RAG-2^−/−^ × RORγt^−/−^ mice compared with CCl_4_-injected RAG-2^−/−^ mice (Fig. 3F). In addition, exogenous IL-22 injection protected RAG-2^−/−^ × RORγt^−/−^ mice from CCl4-induced hepatitis to some extent (Fig. S2).

### Hepatic RORγt^+^ ILCs Are the Main Source of IL-22 Production in CCl_4_-induced Hepatitis in Mice

**Figure 4:**
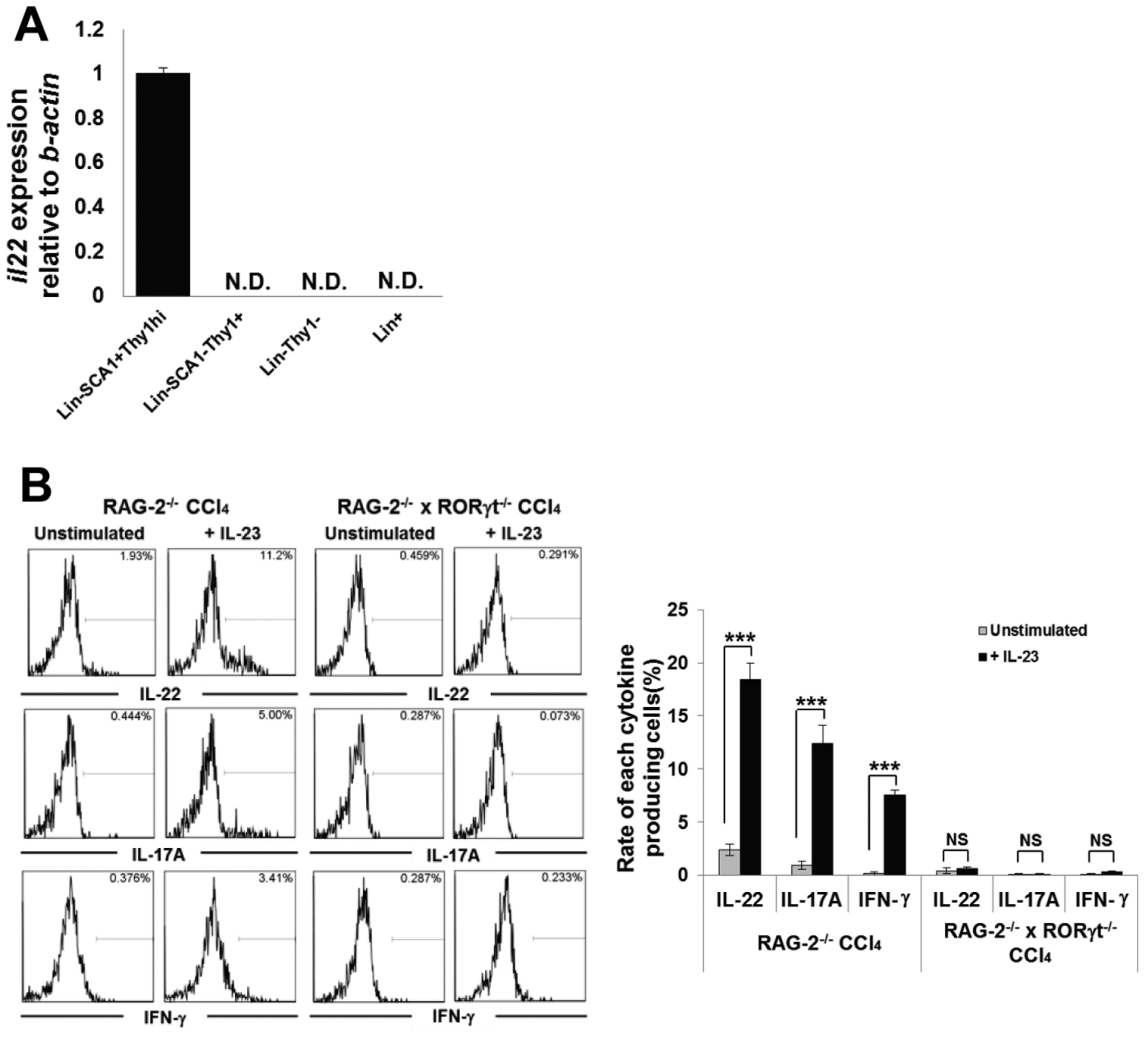
RAG-2^−/−^ × RORγt^−/−^ mice develop more severe CCl_4_-induced hepatitis than RAG-2^−/−^ mice. (A) IL-22 expression in hepatic MNCs of CCl_4_-treated RAG-2^−/−^ mice. IL-22 mRNA expression in the indicated hepatic MNCs subsets was measured by RT-qPCR and normalized relative to β-actin expression. Error bars represent SEM of triplicate samples. (B) Intracellular staining of IL-22, IL-17A and IFN-γ of hepatic ILCs. Hepatic MNCs were collected from RAG-2^−/−^ or RAG-2^−/−^ × RORγt^−/−^ mice 12 h after CCl_4_ injection, and stimulated in the presence or absence of IL-23 for 3 h. Data are representative of eight mice in each group. Mean percentages of cytokine producing cells in the ILC subset. Data show the mean ± SEM (n = 8/group). Data are representative of two independent experiments.

To evaluate the function of hepatic ILCs in the CCl_4_ hepatitis model, we isolated the following MNC subsets 12 h following CCl_4_ administration: Lin^−^SCA-1^+^Thy1^high^ ILCs, Lin^−^SCA-1^−^Thy1^+^ cells, Lin^−^Thy1^−^ cells, Lin^+^ cells, and evaluated the level of IL-22 expression by RT-qPCR. As shown in Fig. 4A, only Lin^−^SCA-1^+^Thy1^high^ ILCs expressed IL-22 after CCl_4_ administration. Furthermore, to investigate cytokines production *in vitro* condition, hepatic MNCs collected from CCl_4_-injected RAG-2^−/−^ or RAG-2^−/−^ × RORγt^−/−^ mice were stimulated with or without IL-23. As the result, fluorescence-activated cell sorter analysis revealed that Lin^−^SCA-1^+^Thy-1^high^ ILCs from CCl_4_-injected RAG-2^−/−^ mice produced IL-22, IL-17A, and IFN-γ upon IL-23 simulation, but those from CCl_4_-injected RAG-2^−/−^ × RORγt^−/−^ mice did not (Fig. 4B). The absence of IL-22 production from hepatic ILCs of CCl_4_-injected RAG-2^−/−^ × RORγt^−/−^ mice *in vitro* was consistent with the result of RT-qPCR analysis of hepatic MNCs isolated from CCl_4_- injected RAG-2^−/−^ × RORγt^−/−^ mice (Fig. 4F).

### Hepatic RORγt^+^ ILCs Are Different from Conventional NK cells which Aren't Related to the Development of CCl4- induced Hepatitis

**Figure 5:**
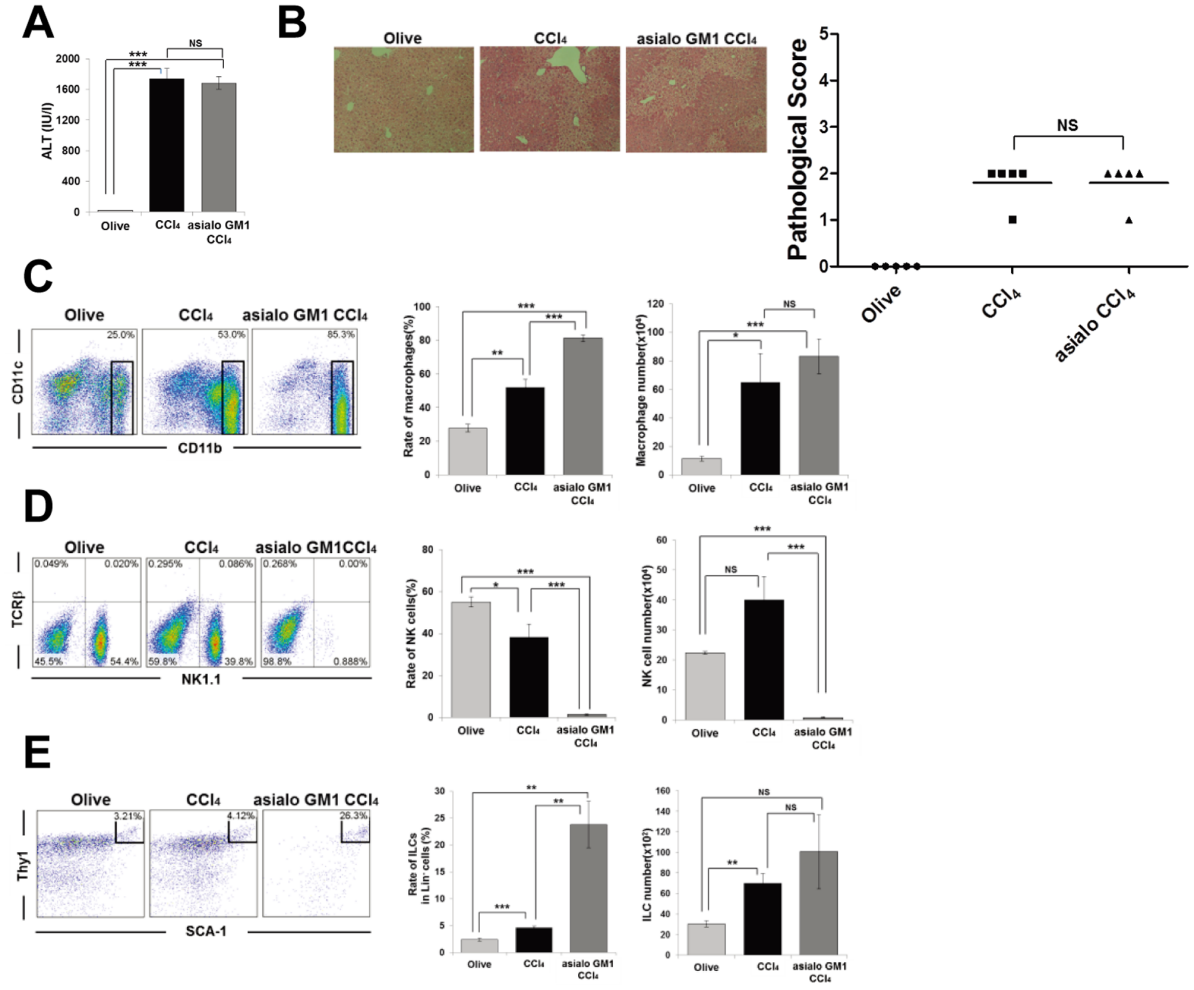
Anti-asialo GM1 Ab treatment does not affect the degree of CCl_4_-induced hepatitis or number of hepatic ILCs. (A) Serum ALT levels of olive oil- or CCl_4_-injected RAG-2^−/−^ mice, or anti-asialo GM1 Ab and CCl_4_ (asialo GM1 CCl_4_) treated RAG-2^−/−^ mice 12 h after CCl_4_ or olive oil injection. Data show the mean ± SEM (n = 5/group). (B) Representative photomicrographs of H&E-stained liver from each group and pathological score. (C) CD11b/CD11c staining of hepatic MNCs from each group 12 h after CCl_4_ or olive oil treatment. Ratio and absolute number of CD11b^+^ macrophages in the hepatic MNCs. Data show the mean ± SEM. (D) TCRβ/NK1.1 staining of hepatic MNCs. Ratio and absolute number of NK cells in the hepatic MNCs. Data show the mean ± SEM. (E) SCA-1/Thy1 staining of Lin^−^ hepatic MNCs. Ratio of ILCs in the Lin^−^ fraction and absolute number of ILCs. Data show the mean ± SEM. Data are representative of two independent experiments.

To further evaluate the possible involvement of NK cells in this model of CCl_4_-induced hepatitis, anti-asialo GM1 Ab was administered to RAG-2^−/−^ mice 1 day and 3 hours prior to before CCl_4_-injection to deplete conventional NK cells. As the result, there was no difference between PBS treated- and anti-asialo GM1 Ab treated- RAG-2^−/−^ mice as to not only the level of serum ALT, but also the degree of histological liver injury (Fig. 5A and 5B). Absolute cell number of macrophages was also not significantly affected by the anti-asialo GM1 Ab treatment (Fig. 5D), although NK cells were efficiently depleted (Fig. 5E), indicating that NK cells are not essential for the development of this model. Furthermore, the injection of anti-asialo GM1 Ab did not deplete hepatic RORγt^+^ ILCs (Fig. 5E). This result suggested that hepatic RORγt^+^ ILCs are different cell population from NK cells.

### Anti-Thy1, but not Anti-NK1.1, mAb Treatment Depletes Hepatic ILCs and Exacerbates CCl_4_-induced Hepatitis

**Figure 6:**
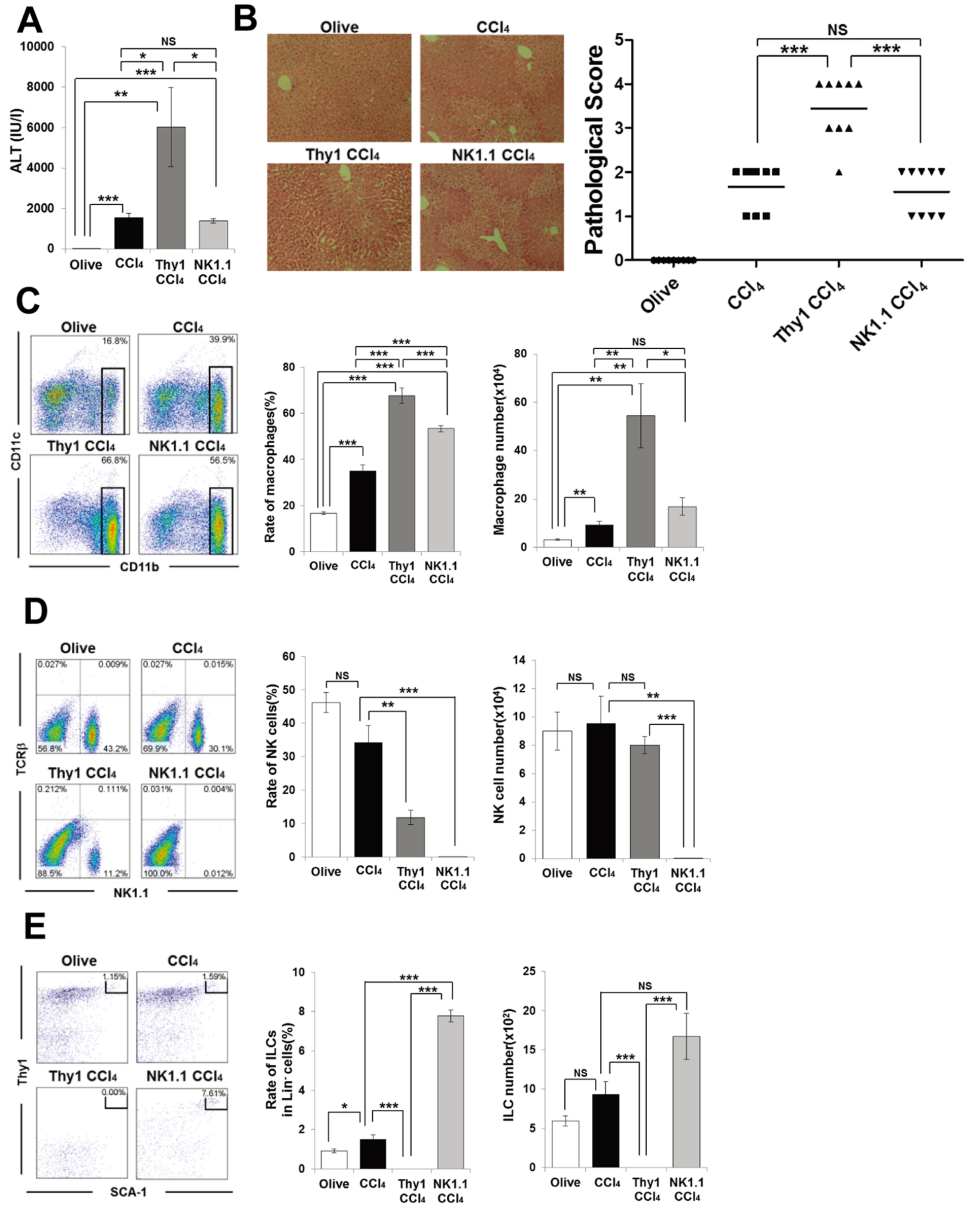
Depletion of ILCs with anti Thy-1 mAb exacerbates CCl_4_-induced hepatitis in RAG-2^−/−^ mice. (A) Serum ALT levels of olive oil or CCl_4_-injected RAG-2^−/−^ mice, or anti-Thy1 mAb and CCl_4_ (Thy1 CCl_4_) or anti-NK1.1 mAb and CCl_4_ (NK1.1 CCl_4_)-treated RAG-2^−/−^ mice 12 h after CCl_4_ or olive oil injection. Data show the mean ± SEM (n = 9/group). (B) Representative photomicrographs of H&E-stained liver from each group and pathological score. (C) CD11b/CD11c staining of hepatic MNCs from each group 12 h after CCl_4_ or olive oil treatment. Ratio and absolute number of CD11b^+^ macrophage in the hepatic MNCs. Data show the mean ± SEM (n = 5/group). (D) TCRβ/NK1.1 staining of hepatic MNCs. Ratio and absolute number of NK cells in the hepatic MNCs. Data show the mean ± SEM. (E) SCA-1/Thy1 staining of Lin^−^ hepatic MNC. Ratio of ILCs in the Lin^−^ fraction and absolute number of ILCs in each group. Data show the mean ± SEM. Data are representative of three independent experiments.

To confirm the novel protective role of RORγt-expressing ILCs in the CCl_4_ hepatitis model, RAG-2^−/−^ mice were treated with anti-Thy1 mAb or anti-NK1.1 mAb to deplete ILCs, as previously shown in murine models of acute colitis [22], and then administered CCl_4_. As shown in Fig. 6A, CCl4-induced serum ALT levels were markedly increased by the anti-Thy1 mAb treatment, but not the anti-NK1.1 mAb treatment. This was concordant with the histological extent of liver damage (Fig. 6B), and an increase in the rate and absolute cell number of macrophages in the liver of anti-Thy1 mAb-treated RAG-2^−/−^ mice (Fig. 6C). Although anti-NK1.1 mAb-treated RAG-2^−/−^ mice had a higher frequency of macrophages, owing to NK cell depletion, there was no difference in the absolute cell numbers among these groups (Fig. 6C). We confirmed that anti-NK1.1 mAb effectively depleted almost all NK cells in the liver, while anti-Thy1 mAb substantially but not completely depleted NK cells (Fig. 6D). Furthermore, in sharp contrast to anti-NK1.1 mAb treatment, ILCs were completely depleted by anti-Thy1 mAb treatment, whereas anti-NK1.1 mAb treatment increased the relative ratio and absolute cell number of ILCs (Fig. 6E). The effects of anti-NK1.1 mAb treatment in RAG2^−/−^ mice were consistent with those of anti-asialo GM1 Ab that depletes conventional NK cells (Fig. 5).

## Discussion

**Figure 7:**
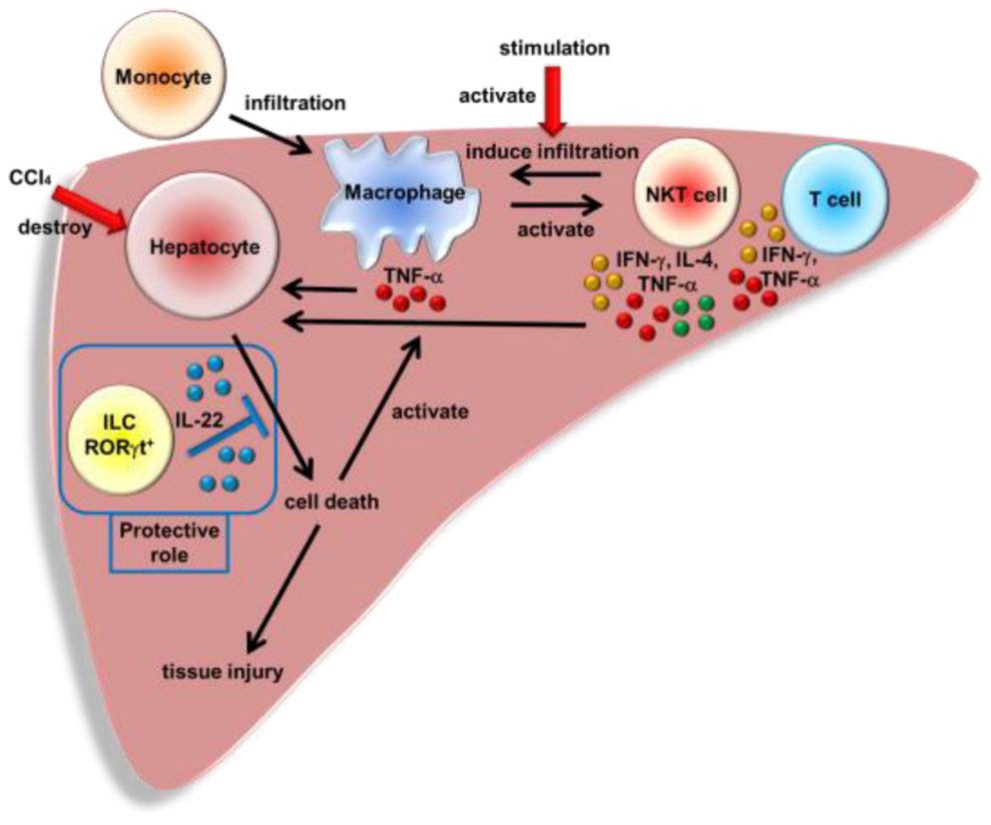
Protective role of RORγt-dependent hepatic ILCs in acute hepatitis. RORγt-dependent hepatic ILCs attenuate acute liver injury through the production of IL-22.

The current study demonstrated that RORγt-dependent hepatic ILCs played a protective role on murine acute liver injury in IL-22 dependent manner. Fig. 7 gives a schematic view of the proposed roles of RORγt-dependent hepatic ILCs in acute hepatitis models revealed in this study.

Previous reports have demonstrated the importance of RORγt-dependent cells in various immune responses, as RORγt is essential for the development of lymphoid tissues and innate and adaptive immune cells such as Th17 cells, invariant or IL-17A producing NKT cells and ILCs [1], [2], [10], [11], [17], [23]. To investigate the roles of RORγt-dependent cells in the liver, we focused on RORγt expression in hepatic MNCs subsets. We found that hepatic ILCs highly expressed RORγt in addition to CD4^+^ T cells and NKT cells, and the number of hepatic ILC cells was considerably decreased in the liver of RAG-2^−/−^ × RORγt^−/−^ mice. This suggests the possibility that RORγt-dependent hepatic ILCs are involved in hepatic immune responses. Notably, previous reports have emphasized that IL-22 produced by Th17 cells can act protectively in various acute hepatitis models, although NKT cells are pathogenic in the development of Con A-induced hepatitis [13], [16]. Furthermore, we found that stimulated NKT cells induced the accumulation of inflammatory macrophages in the liver.

To investigate the roles of RORγt-dependent cells in the pathogenesis of acute liver injury, we first performed CCl_4_- induced acute hepatitis model in WT and RORγt^−/−^ mice. Administration of a single dose of CCl_4_, characterized by the accumulation of macrophages and NKT cells, [19], [21] caused a severe acute hepatitis both in WT and RORγt^−/−^ mice. Development of a similar degree of hepatitis following CCl_4_ administration irrespective of the significant decrease in the number of macrophages and NKT cells in the injured liver of CCl_4_-injected RORγt^−/−^ mice compared with WT mice indicated that some RORγt-dependent pathological and protective immune cells keep a delicate balance in this model. As a candidate of RORγt-dependent protective cells, we focused on hepatic ILCs that highly express RORγt. In this regard, a Lin^−^SCA-1^+^Thy1^high^ ILC subset expressing IL-17A, IFN-γ, and IL-22 was shown to reside in the liver of *H. hepaticus*-infected mice, but their functional roles were not determined [2], [22]. We identified the existence of these hepatic cells also in the normal condition without *H. hepaticus*-infection, and clarified that they have a similar feature with previously reported intestinal ILCs as to the expression of some surface markers [2]. We next performed CCl_4_- induced acute hepatitis model using RORγt^−/−^ and RAG-2^−/−^ × RORγt^−/−^ mice to investigate the roles of RORγt-dependent cells in the liver except Th17 cells and NKT cells. As expected, CCl_4_-adminstered RAG2^−/−^ × RORγt^−/−^ mice showed a severer hepatitis compared with CCl_4_-adminstered RAG-2^−/−^ mice. Interestingly, hepatic MNCs from CCl_4_-treated RAG-2^−/−^ mice consistently expressed IL-22, although they lack Th17 cells previously known as the source of IL-22 in the development of murine acute hepatitis models [7], [9]. On the other hand, hepatic MNCs from CCl_4_-treated RAG-2^−/−^ × RORγt^−/−^ mice lacked IL-22 expression. Supporting these results, only Lin^−^SCA-1^+^Thy1^high^ hepatic ILCs expressed IL-22 in the liver CCl_4_-treated RAG-2^−/−^ mice, and hepatic ILCs obtained from the liver of CCl_4_-treated RAG-2^−/−^ mice, but not those from CCl_4_-treated RAG-2^−/−^ × RORγt^−/−^ mice, produced IL-22 in response to IL-23 *in vitro*. Furthermore, the number of RORγt^+^ hepatic ILCs was also decreased, and exogenous IL-22 administration protected RAG-2^−/−^ × RORγt^−/−^ mice from hepatitis. This suggests a novel protective function of IL-22-expressing hepatic ILCs against acute liver injury. This was also confirmed using depleting mAbs. Hepatic RORγt^+^ ILCs were depleted by anti-Thy1 mAb, but not by anti-NK1.1 mAb or anti-asialo GM1 Ab, in the liver of CCl_4_-administrated RAG-2^−/−^ mice causing a severer hepatitis. These results indicated that hepatic RORγt^+^ ILCs are different from conventional NK cells and act protectively against liver injury. Although hepatic RORγt^+^ ILCs produce IFN-γ which is one of the pathological factors in CCl4-induced hepatitis, [24] that function of hepatic RORγt^+^ ILCs seems to be not critical for the development of hepatitis since there was no difference between the livers from CCl_4_-administrated RAG-2^−/−^ mice and RAG-2^−/−^ × RORγt^−/−^ mice in terms of the expression of IFN-γ.

We also noticed that RAG-2^−/−^ × RORγt^−/−^ mice developed severer hepatitis following CCl_4_ administration when compared with RORγt^−/−^ mice. These results may indicate that other tissue-protective cell subsets in B cells, T cells and/or NKT cells can ameliorate CCl_4_-induced tissue damage, although further study is needed to address this issue in the future.

The functions of some hepatic immunocompetent cells, such as T cells, NKT cells and macrophages have been believed to include defense against microbial pathogens or viral infection in the liver, [25]–[27] but can also inhibit the growth of cancer [28]. These cells mediate these functions by secreting inflammatory cytokines, like IFN-γ and TNF-α, to induce cytotoxicity in infected cells and tumor cells [25]–[28]. Accordingly, those inflammatory cells also induce liver injury when activated excessively, and cause acute hepatitis as shown in some murine acute hepatitis models [19], [29]. IL-22 produced by RORγt^+^ hepatic ILCs may counteract inflammatory cells-mediated liver injury because IL-22 is a survival factor for hepatocytes by preventing and repairing liver damage [7]. RORγt is critically involved in the development and function of hepatic RORγt^+^ ILCs, also is important for Th17 cell development [1], [2], [4], [10], [11], [17], thus Th17 cells may also play a role in the pathogenesis of liver inflammation, although this is still controversial [9], [30], [31]. However, as to the source of IL-22, Th17 cells certainly have a protective role against liver injury [9], [23]. Although hepatic RORγt^+^ ILCs also have an ability to produce IL-22, hepatic RORγt^+^ ILCs may be able to act at an early innate immune response stage of liver injury compared with Th17 cells since they exist also in the naïve condition.

We conclude that RORγt^+^ hepatic ILCs have a pivotal protective function against liver injury via IL-22 production. Therefore, RORγt^+^ hepatic ILCs may be involved in clinical hepatitis and represent a candidate for a target of novel treatment for clinical hepatitis since IL-22 can prevent and repair liver damage.

## Supporting Information

Figure S1 **RORγt^−/−^ mice are resistant to αGalCer-induced hepatitis.** (A) Serum ALT levels of WT or RORγt^−/−^ mice 12 h after αGalCer or PBS injection. Data show the mean ± SEM (n = 5/group,). (B) Representative photomicrographs of H&E-stained sections of the liver from each group. (C) CD11b/CD11c staining of hepatic MNCs from WT or RORγt^−/−^ mice 12 h after αGalCer or PBS injection. Ratio and absolute number of CD11b^+^ macrophage in the hepatic MNCs. Data show the mean ± SEM (n = 5/group). (D) TCRβ and NK1.1 staining of hepatic MNCs. Percentage and absolute number of NKT cells in the hepatic MNCs. Data show the mean ± SEM (n = 5/group). Data are representative of four independent experiments.
(TIF)

Figure S2 **Exogenous IL-22 administration protects RAG-2^−/−^ × RORγt^−/−^ mice from CCl_4_-induced hepatitis.** (A) Serum ALT levels of RAG-2^−/−^ × RORγt^−/−^ mice 12 h after CCl_4_ or IL-22 and CCl_4_ injection (n = 7/group). Data show the mean ± SEM. (B) Representative photomicrographs of H&E-stained liver from each group and pathological score. (C) CD11b/CD11c staining of hepatic MNCs from RAG-2^−/−^ × RORγt^−/−^ mice 12 h after CCl_4_ or IL-22 and CCl_4_ treatment. Ratio and absolute number of CD11b^+^ macrophages in the hepatic MNCs. Data show the mean ± SEM. Data are representative of two independent experiments.
(TIF)
