# Supplementary figures and images for: IL-22-Producing RORγt-Dependent Innate Lymphoid Cells Play a Novel Protective Role in Murine Acute Hepatitis
Source: PLoS One. 2013 Apr 23;8(4):e62853. doi: 10.1371/journal.pone.0062853 (PMC3633830; doi:10.1371/journal.pone.0062853)

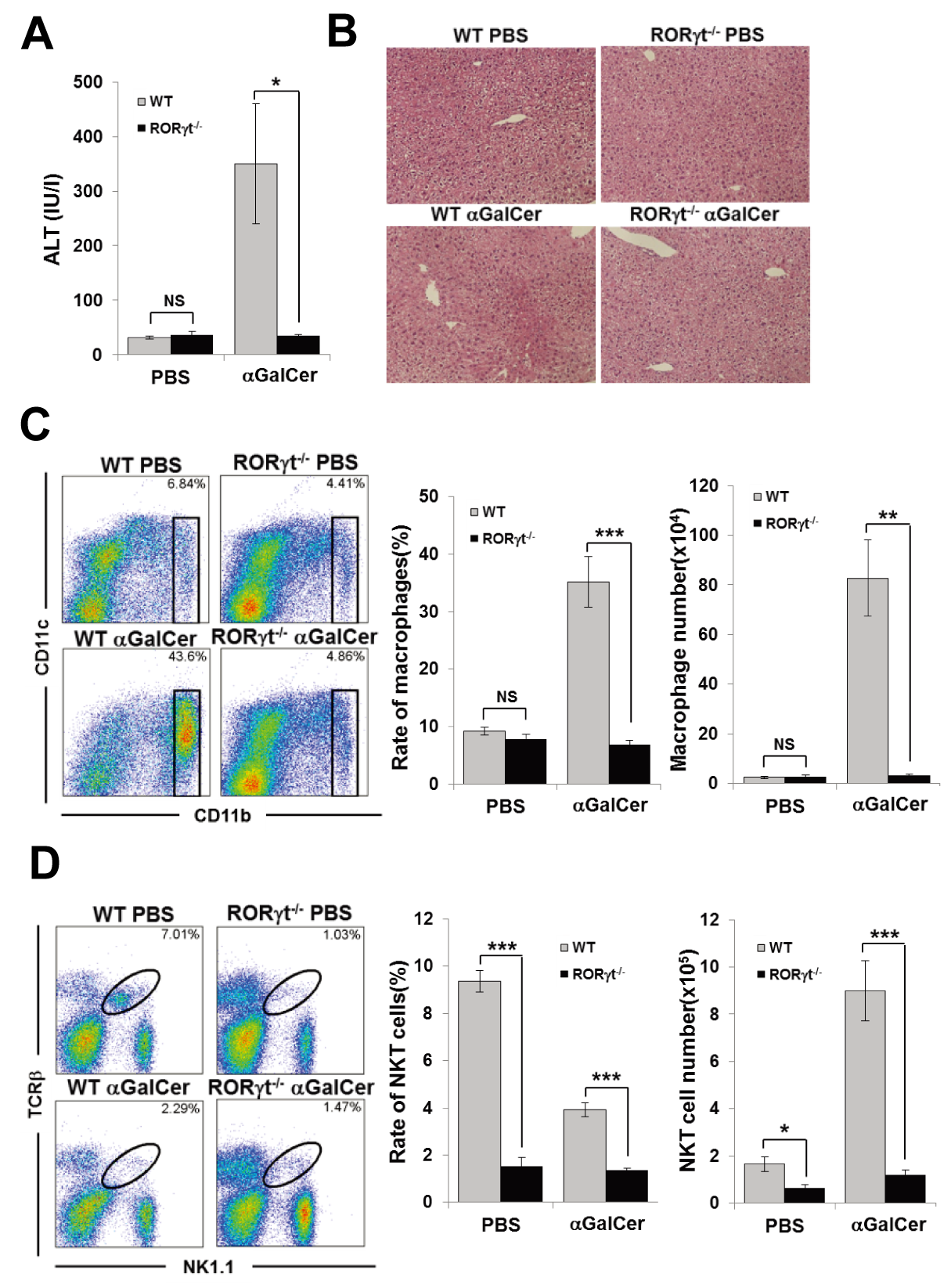

Supplement: Figure S1 — RORγt−/− mice are resistant to αGalCer-induced hepatitis. (A) Serum ALT levels of WT or RORγt−/− mice 12 h after αGalCer or PBS injection. Data show the mean ± SEM (n = 5/group,). (B) Representative photomicrographs of H&E-stained sections of the liver from each group. (C) CD11b/CD11c staining of hepatic MNCs from WT or RORγt−/− mice 12 h after αGalCer or PBS injection. Ratio and absolute number of CD11b+ macrophage in the hepatic MNCs. Data show the mean ± SEM (n = 5/group). (D) TCRβ and NK1.1 staining of hepatic MNCs. Percentage and absolute number of NKT cells in the hepatic MNCs. Data show the mean ± SEM (n = 5/group). Data are representative of four independent experiments. (TIF) [file pone.0062853.s001.tif]

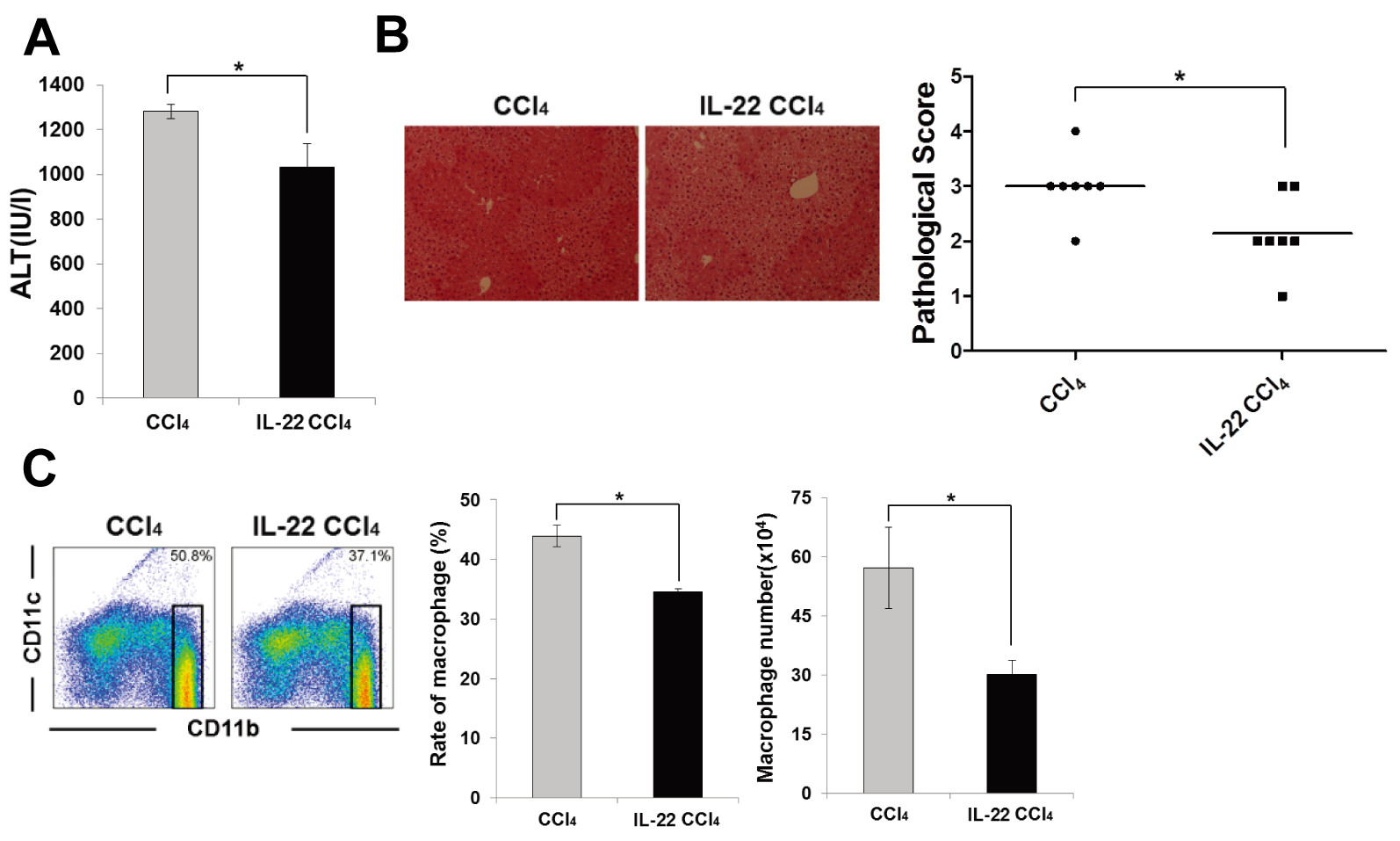

Supplement: Figure S2 — Exogenous IL-22 administration protects RAG-2−/− × RORγt−/− mice from CCl4-induced hepatitis. (A) Serum ALT levels of RAG-2−/− × RORγt−/− mice 12 h after CCl4 or IL-22 and CCl4 injection (n = 7/group). Data show the mean ± SEM. (B) Representative photomicrographs of H&E-stained liver from each group and pathological score. (C) CD11b/CD11c staining of hepatic MNCs from RAG-2−/− × RORγt−/− mice 12 h after CCl4 or IL-22 and CCl4 treatment. Ratio and absolute number of CD11b+ macrophages in the hepatic MNCs. Data show the mean ± SEM. Data are representative of two independent experiments. (TIF) [file pone.0062853.s002.tif]
